# Atlanta Medical College Clinic

**Published:** 1859-02

**Authors:** W. F. Westmoreland


ATLANTA

Medical and Surgical Journal.

Vol. IV.] FEBRUARY, 1859. [No. VI.

### ORIGINAL COMMUNICATIONS.

### ARTICLE I.

*Atlanta Medical College Clinic*—November 9th, 1858.
Service of Dr. W. F. Westmoreland.

*Secondary Syphilis.*—Caroline, servant, aged 22, good constitution. Has been, for three months or more, troubled with an eruption upon the head, face, neck and chest—ulcers upon the tonsils, tongue and mucous membrane of the lips. Denies positively ever having had a chancre, but from the appearance of the mucous ulcerations, the eruption upon the face, &c., as well as an enlargement of the superficial cervical glands posterior to the insertion of the sterno mastoid muscles, there can be but little doubt as to the character of the affection.

The enlargement of the above mentioned glands should be regarded as an important diagnostic sign, as there are but very few cases of Secondary Syphilis, in which one or more of this group may not be found just beneath the skin, giving somewhat the feeling of a bullet or shot imbedded in the tissues.

In the treatment of this form of Syphilis, there are some who rely almost solely upon some of the preparations of Mercury, while others exclude them entirely. With the latter, the Iodide of Potassium may be placed at the head of the list.

Mercury, when not contra-indicated, as in the Scrofulous diathesis, for example, is certainly the most efficient, but should always be used with care. Profuse ptyalism is always objectionable, and may result in unpleasant consequences, without in the least hastening the cure. Great difference of opinion exists as to the most efficient preparation of Mercury. Ricord's favorite is the Protiodide of Mercury.

Caroline will take the following: ℞. Pil. Hidrarg. ʒi.; Opii Pulv. grs. v.—Make twelve pills, one to be taken morning and night, with a wine-glassfull of the decoction of the bark of the root of the Juglans Nigre.

*Fistula in ano, with Induration or Stricture of the rectum.*—William, servant, aged 24, has suffered for two years with disease of the rectum and anus; has been submitted to various plans of treatment—had two operations performed before coming to Atlanta. He first presented himself at the College Clinic four months ago, with the following symptoms:

Three or four hemorrhoidal tumors of considerable size, surrounded the anus; copious discharges of a sero-pus, sometimes mixed with blood, both from the anus and a fistulus opening communicating with the rectum; a director could be readily passed through the fistulous opening into the rectum; has suffered no great pain at any time; considerable induration of the rectum about the internal sphincter. The hemorrhoidal tumors were removed a few days after the first examination, with the Ecraseur, without the loss of the smallest quantity of blood; ten days or two weeks later, the operation for fistula in ano was performed, and the wound filled with lint. The fistulous track filled readily with healthy granulations, so that at the expiration of two weeks the wound had healed. But notwithstanding the obliteration of the fistulous track, the discharge was but slightly lessened. Upon a more extended examination, the rectum was found greatly indurated, as high as could be reached with the finger. There was not only a thickening of the mucous membrane, but apparently a deposit in or between the other coats of the bowel. The speculum revealed several large ulcers, which would bleed from the slightest touch. A solution of the nitrate of silver, ten grains to the ounce was used as injection, and later, a saturated solution without any marked change.

The Tinct. of Iodine, has also been used in the same way and with some advantage. As this condition of the rectum is sometimes induced by a syphilitic taint, the Iodideof Potassium was used in large doses for a length of time, without any perceptible effect. There is at present considerable thickening of the walls of the rectum. Some two inches and a half above the anus, the stricture is so great, the index finger cannot be passed without great pain to the patient. The discharge, which is to a very great extent, from the ulcers and abrasions, is much less in quantity than some weeks past.

TREATMENT.—Continue the injections of the Tinct. Iodine. Daily introduction of rectal bougies, with the object of enlarging the canal.

*Secondary Syphilis with Syphilitic Iritis.*—Green, servant, aged 47—had a chancre, some three months ago, which disappeared in a short time. Two or three weeks after, there appeared an eruption upon the face, head, neck, chest, and abdomen, abrasions upon the buccal mucous membrane, with an enlargement of the posterior cervical glands. Considerable induration around the cicatrix, from the chancre, right eye considerably inflamed, for the relief of which, he was induced to visit the College Clinic. Slight change of color in the iris of the right eye—pupil contracted, rather oval than round, cloudy, with a deposit of fibrin upon the border of the iris forming the pupil. Considerable inflammation of the conjunctiva, which is certainly secondary, the inflammation having extended from the iris.

Green has taken several remedies for the eruption, without, however, suspecting its character; and for the inflamed eye, has applied poultices, used astringent washes, &c., but without benefit, as might have been expected.

*Treatment.*—As there is no doubt of the specific character of both the eruption and the iritis, the patient should be put upon anti-syphilitic remedies. The following is perhaps the very best, as mercurials are always important where iritis is a prominent symptom: ℞. Pil. Hydrarg. ʒi; Opii Pulv. grs. iv. Make twelve pills—one morning and night. Wine-glassfull of the decoction of the bark of the root of the Juglans Nigre, three times a day. For the iritis, cups to the right temple two or three ounces; plaster of the extract of Belladonna, 2 by 2, over the right eye until the pupil is dilated. The dilatation of the pupil, in iritis, is of the very greatest importance, as it breaks up adhesions, the result of the effusion of plastic lymph, which is very perceptible in this case, and in this way prevents the obliteration of the pupil.

NOVEMBER 10th.—*Fracture of the Nasal Bones.*—Charles B——, aged 14, received a blow two days ago upon the nose, producing a compound fracture of the nasal bones; right nasal bone considerably depressed. A director was introduced into the nostril, and the depressed bone restored to its normal position. As there was no tendency to displacement after the reduction, nothing was placed in the nostril to sustain the fragment. The wound in the skin was brought together with adhesive plaster, and the patient requested to call again in a few days.

*Hereditary Syphilis.*—Augusta, (mulatto) aged 7, delicate. Four or five weeks ago complained of pain over the temporal region, which was, at the time, regarded as neuralgic; two weeks later, some tumefaction was perceptible over the inferior border of the parietal bone, which continued to increase until a few days ago, when from fluctuation in the tumor, it was evident that there was an accumulation of pus. An incision procured a rather copious discharge of an unhealthy looking sero-pus—the discharge still continues. The introduction of a probe revealed the fact that there was caries of the parietal bone, and that the abscess was the result of the disease of the bone. Two other small tumors were found over the cranial bones at other points. From the symptoms present, as well as the fact that the father has had syphilis, there is but little doubt that all depends upon a syphilitic taint. The treatment of hereditary syphilis differs but little, if any, from the tertiary form of this disease, when the bones are attacked. This little girl, then, will take the following prescription: ℞. Syrup of Gentian, ℥vii; Iodide of Potassium, ʒvij. Dissolve—take a teaspoonful three times a day before meals, the dose to be gradually increased until she takes thirty grains a day. As the condition of the system requires a tonic, perhaps the very best that could be used in this case is the Iodide of Iron. She will take then the Syrup of the Iodide of Iron in doses of ten drops three times a day. The caries, with the congestion of the soft parts consequent upon this condition of the bone, would, perhaps, be benefitted by injecting into the abscess the Tinct. of Iodine every second or third day.

*Valvular Disease of the Heart.*—Mr. S——, teacher, aged 45; some years ago suffered slightly from rheumatism; for a year or two past, has been troubled with palpitation of the heart, dyspnœa, and, in fact, that train of symptoms which usually accompany organic disease of the heart. The pulsations of the heart rather irregular—a blowing or bellows murmur accompanying the first sound of the heart—maximum at the apex, evidently indicating a lesion of the mitral or tricuspid valves, or both. Complains also of indigestion, which increases his cardiac disturbance. Permanent relief in such cases should not be expected, but in a number, much may be done towards relieving the sufferings of the patient, and staying the fatal day.

Such remedies as will reduce the frequency and force of the heart's action will often relieve the palpitation, dyspnœa, &c., and render the patient much more comfortable. The Tinct. of Digitalis stands at the head of the list of this class of remedies.

*Treatment.*—Tinct. Digitalis, seven drops, three times a day; the dose to be gradually increased to ten drops.

For the indigestion, a wine glass full of a strong decoction of equal portions of Columbo and Gentian, three times a day.

NOVEMBER 11th.—*Fistula in Ano.*—Mr. T——, aged 69, was operated upon in 1855 for fistula in ano with success; has had no symptoms of a return of the disease until three or four weeks ago. At that time a small abscess formed in the cicatrix, from the wound by the operation, and bursted externally. Since that time there has been a copious discharge from the fistulous opening. A careful examination failed to find an opening into the rectum. The fistula then is incomplete—*blind external fistula.* The Tinct. Iodine was injected into the fistulous opening—to be repeated every 3d day.

NOVEMBER 14th.—*Pulmonary Emphysema with Asthma.*—Mr. J——, aged 35, has suffered for two or three years past with frequent attacks of asthma; constant cough without much expectoration. Upon percussion over the anterior portion of the chest, the sound is morbidly clear; auscultation revealed a feeble or suppressed respiratory murmur in the same locality; sounds of the heart normal, but distant and suppressed as if there was some intervening porous substance between that organ and the ear. (In this disease there is an augmentation of the size of the air vesicles, sometimes reaching the size of a cherry stone; in other cases there is a rupture of the air vesicles, half dozen or more communicating, and if near the surface of the lung forming a projection or tumor. In other cases there is rupture of the air vesicles, and an infiltration into the interlobular cellular tissue.) Has suffered from indigestion; great constipation of the bowels; has been in the habit of taking cathartic pills every few days. The paroxysms of asthma in this case are the result of the condition of the lung above described.

*Treatment.*—Stimulants and anodynes to prevent or shorten the paroxysms; fully impressed with the opinion that the sufferings of the patient are greatly increased by the condition of the stomach and bowels; the remedies at present should be directed more particularly to those organs.

A strong decoction of equal portions of Columbo and Gentian, wine glass full three times a day, should be taken. For the costive habit, regularity in going to stool—the same hour every day should be selected—and if not successful in relieving the bowel of its contents, enemas of cold water should be used at the time, in sufficient quantity to induce the contraction of the bowel.

NOVEMBER 15th.—*Lumbar Abscess.*—David (servant) aged 24; has been sick two years; was first attacked with pain in the back, and soon after, two or three tumors made their appearance in the region of the lumbar and dorsal vertebræ. Some months after the appearance of the tumors in the lumbar region, another made its appearance upon the external surface of the thigh, an inch-and-a-half or two inches below the trochanter major, and a very short time after, another appeared upon the internal surface of the thigh just below Pouparts ligament. The tumors in the lumbar region diminished in size as those in the thigh enlarged, so that the tumors in that region are at present hardly perceptible.

Twelve or fourteen months ago, the cervical glands commenced to enlarge, and upon the left side, the induration was immense, presenting a tumor the size of the first or larger: the glands suppurated, forming an extensive abscess which was opened a month or two ago, and is now very nearly well.

The tumors upon the external and internal surface of the thigh, are at present of considerable size. Fluctuation is perceptible. There is evidently a communication between them, as the liquid in one may be forced into the other.

These tumors, or rather abscesses, as they contain pus, as will be shown directly, are the result of the disease of the vertebræ; the pus was formed at the carious point or points of the vertebral column, and by distension dissected up the fascia, and following the track of the psoas magnus and psoas parvus muscles, reached the thigh. There is no perceptible deviation of the spinal column, but great tenderness and pain in the lumbar and dorsal regions.

The patient, for months past, has been upon tonic, nourishing diet, and various other remedies have been administered with the view, more particularly, of correcting the scrofulous diathesis, which certainly exists in this case. In removing this extensive accumulation of pus, care should be had that air does not enter the cyst which contains the fluid, else serious and even fatal accidents might arise. To prevent such accidents the plan suggested by M. Chassaignac was adopted. The abscess was transfixed by a long trocar and canula, the trocar then removed, and a small india rubber tube, perforated by numerous small openings, was introduced through the canula, and the canula then removed, thus leaving the tube in the position occupied by the canula. The abscess is thus transfixed by the tube, the pus, as soon as formed, entering the tube through the openings alluded to, and passing off. The iodide of potassium, cod liver oil, syrup of the iodide of iron, &c., should be administered when circumstances will permit.

NOVEMBER 16th.—*Secondary Syphilis.*—Melinda, mulatto, aged 18. stout, has had an eruption for two or three weeks upon the head, face, and trunk. Small ulcers upon the buccal mucous membrane; mucous ulcerations upon the vulva; had a chancre two months ago. The administration of mercury for Secondary Syphilis in the mulatto, is, in a number of cases, of doubtful propriety. This class of subjects, above all others, are predisposed to scrofula, and, perhaps, in the majority of cases, if it does not actually exist, there is a predisposition to the disease. As Melinda was stout, and apparently of a most excellent constitution, that preparation of mercury, which has been so often prescribed here in similar cases, was suggested: ℞. Pil. Hydrag. ʒi; Opii. Pulv. grs. v. Make twelve pills—one night and morning. A wine-glassfull of a decoction of the bark of the root of the black walnut, three times a day.

*Scrofulous Ulceration of the Cornea.*—Betsey, aged 7, large abdomen, emaciated extremities, induration of the lymphatic glands, superior lip large, apparently tumefied—the type of scrofulus diathesis. For more than a year past the right eye has been inflamed, with considerable tumefaction of profuse secretion, which produced ulcers and abrasions of the skin over which the secretions passed. There are large ulcers upon the inferior portion of the cornea; and numerous large vessels extending from the conjunctiva over the cornea to the ulcers.

Treatment:—Section of the vessels alluded to, at the junction of the cornea and sclerotic, by means of a curved or convex scarificator, made for the purpose, the incision sufficient to make a complete section of the conjunctiva, at that point. As a wash for the eye there is perhaps nothing better than a weak solution of the sulphate of zinc: ℞. Sulphate of Zinc, grs. iij; Distilled Water, ℥ij. dissolve. A few drops introduced into the eye, two or three times a day. Extreme cleanliness is important, and after each cleansing, the lids and face, where the secretions pass, should be covered with simple cerate, sweet oil, or some other unctuous substance to prevent the irritating effect of the secretions. The cauterization of the ulcers with nitrate of silver would be extremely hazardous, as we might produce a slough by such application as would perforate the cornea, thus obliterating the anterior chamber, producing hernia of the iris and other accidents which might result in the complete destruction of the eye.

The constitutional treatment is very important, as all depends upon the constitutional defect. Betsey, then, will take syrup of the iodide of iron, ten drops three times a day, iodide of potassium in five grain doses, three times a day, in combination with some vegetable tonic, perhaps equal parts of the tincture of columbo and gentian—nourishing diet is very important.

---

*Service of Dr. Brown.*

*Tuberculosis.*—Mary, servant girl, age 15 or 16 years, presented herself, complaining of occasional pains in the chest, upper part; cough more troublesome at night, with excessive muco-purulent expectoration; general debility, and retention of menses. Says that 3 years since, she had scarlet fever ; was previously well, and had menstrual flow—normally established. After the scarlet fever, a short while, cough made its appearance, and continued to increase upon her in spite of all treatment. She has had no recurrence since, of the uterine secretion.

On examination, find the digestive apparatus normal—mammary glands scarcely at all developed—auscultation and percussion disclose the seat of greatest difficulty. We have tubercular deposit in apex of right lung ; perhaps some on left side. Bronchitis of long continuance, and consequent enlargement of bronchial tubes, with bronchophony. We gain nothing by external examination of uterine region. The proximate cause of the retention of catamenia, supposed to be tuberculosis. The remote and primitive cause of all her troubles, scarlatina. The ravages of this disease upon the tissues, are too often witnessed in the various sequela, as they are called, depressing the vital powers to such an extent, in the effort to eliminate, perhaps as to invite, if not develop many functional and organic diseases, of which we have here an instance.

In the treatment, let us endeavor to improve the character of the blood ; impart tone and vigor to the various tissues, that we may stay, if possible, this deposit in the lungs ; leaving the uterus to follow in the train of healthy action. Inhalation of vapor of iodine every few days, nourishing and oily food, with alcoholic drinks taken moderately after eating ; exercise in the open air, with good, comfortable clothing, may, perhaps, tend to accomplish this.

*Varicose Veins.*—Mr. H. presents himself, about 60 years of age, a native of Ireland—a laborer—his constitution much impaired by age and irregular habits ; is troubled with varicose veins, in the leg, with consequent abrasion of surface over the Tibia.—His troubles are doubtless, immediately, caused by weakness in the coats of the veins here. This state of things may exist with or without disease of the venous coats—constitutional peculiarities in that system of vessels ; long continued pressure from the column of blood ; general debility, and consequent loss of contractility in the vessels of part ; exposure and injuries received here, may all be causes of this troublesome affection. Treatment, age and habits considered, we can only hope to palliate.—Paint the parts, around the abrasion, with *Tincture Iodine;* apply constantly the Roller Bandage, protecting the ulcerating points by oil silk; tonics with generous diet and temperance.

*Conjunctivitis*—George, servant boy, six or eight years old; mulatto or mixed blood; Conjunctivitis; somewhat chronic in its character; slight opacity of the cornea, "nebula."

The result, it is supposed, of long continued congestion during paroxysms of hooping cough; sometimes followed by well marked convulsions, frail and delicate constitution. Treatment—Blister to nape of neck. to be kept up by renewal or irritants; tonics and alteratives. ℞ Com. Tinct. Gentian, ℥iv; Hydriodate Potassia, ʒii; Dose, 1 to 2 ʒ 3 times per day; astringent collyriam, nourishing diet, &c.

*Otirrhea.*—M. A——; truly interesting case, the like of which you will often, perhaps, meet in after years Obstinate and intractable as the disease often is, it is highly important that we understand it properly. The patient is about ten years of age, has rather a healthy appearance, but here you see the long, weeping eyelash, transparent skin, and somewhat pouting lip, &c., which, to my mind, strongly indicate the Scrofulous Diathesis; she has otitis with otirrhœa, severe headache occasionally, slight cold increases the discharge from the ear, and induces headache more severe than in ordinary children. From slight fatigue she complains much, and has frequently following, heavy colliquative night sweats. Sometime since she had scarlet fever; that scourge of children had here favorable ground to do its work of disorganization: a scrofulous constitution. The eruption came out imperfectly; following the period of desquamation, came the discharge from ear, and has since continued.

Treatment: Here again we must effect a change and improvement in the character of the blood, to increase the vigor of constitution. Hydriodate of Potassa, with Tincture Gentian or other tonic; local injection to ear, of Tincture Iodine dilute, with the hope of stimulating or arousing the tissue involved, to a more healthy action; nutritious food, warm clothing, and plenty of exercise in open air.

*Stricture.*—Mr. C—— presents himself with Stricture Urethræ; following case of Gonorrhœa; situated as you see, byuse of bougie, in the membranous portion---varieties given,---shall endeavor to overcome it by use of bougies entirely, grad ually increasing in size of the instrument, and daily introduced as you shall see.

*Conjunctivitis.*---Mr. B——. A case of conjunctivitis, with ulceration of cornea, following attack of measles; reports himself better, under treatment before directed, viz; Collyrium of Sul. Zinc Solution, Cups to the temple at night; Blue Mass 5 grs. Continued to improve.

*Dyspepsia.*---Mrs. R——, sixty-three years of age, has Dyspepsia, with constipation, &c., great smoker. Treatment, palliative; leave off the pipe; give Com. Tinc Aloes, ʒi thrice daily, increased or lessened according to effect on bowels, &c.

Upon a subsequent occasion, several of the patients previously prescribed for, presented themselves. Mr. B. with Conjunctivitis improved, and a continuation of the treatment directed.

Mr. H. with Varicose Veins much improved, and the same treatment continued.

Mrs. R. with Dyspepsia, no improvement. Same treatment, with the addition of Sulp. Ferre gr. i in pill or solution 3 times daily.

Miss M., with Otirrhœa, decidedly benefitted by previous prescription---continuation of the same.

---

*Service of Dr. Powell.*

NOVEMBER 14—*Ulceration of the Os Uteri.*—Betsy, negro, dark complexion, age 28 or 30; is the mother of four children, enjoyed good health up to the birth of her last child, which is four years old, since which time her health has been declining; has suffered more or less from the following symptoms: Pain in the hips and back, extending frequently to the anterior parts of the thighs. She sleeps badly, is very nervous, now and then amounting to hysteria; bowels rather loose since she had the Measles last Spring. Has headache frequently, catamenia regular, but attended with a great deal of pain. Skin dry and ashy color, pulse rather quick and irritable. Urine high-colored and gives pain in passing.

An examination by the touch, revealed the os enlarged and indurated. The speculum was introduced, which brought to view the enlarged, dilated and indurated os; indeed, the entire cervix was enlarged, and upon the posterior lip, is situated a large dark ulcer.

*Treatment.*—Cauterized the ulcer freely with solid nitrate of silver, passing the stick into the canal, and applying it to every accessible point. A vaginal injection morning and night, of a saturated solution of Chlorate of Potassa, and take one tea-spoonful three times a day, commencing ten days before the menstrual discharge, of the following: ℞. Prep. Sulphur ʒii; Gum Guiac ℥i; Brandy oi.

NOVEMBER 29—*Inflammation, &c., of Cervix Uteri.*—Charlotte, Mulatto, thirty-eight or forty years of age, strumous diathesis, rather tall and inclined to emaciation. She began to menstrate at 14 or 16 years of age, and was very regular up to the birth of her first and only child, which was about 16 years ago, since which time the menstral function has been irregularly performed, and always attended with much pain. Her present symptoms are frequent severe headache, palpitation, dyspnea, cough, pain in the back and lower part of the abdomen, particularly in the right side, with pelvic weight and bearing down. She has also for the last two years, been troubled with a constant yellowish white discharge, occasionally much streaked with blood. The abdominal muscles also contract from spinal irritation, and assume the appearance of a tumor, which is by no means unusual in uterine diseases. She has lost her appetite, and her general health has given way very much. A vaginal examination was made before the Class with the speculum; the uterus was found slightly prolapsed; no ulceration, but soft engorgement of the neck; the slightest pressure by the instrument, caused drops of blood to exude from its surface; also a thick mucous discharge from the os.

*Treatment.*—For a few days, cold mucilaginous injections three times a day, and afterwards a saturated solution of chlorate of potassa, and at the same time to secure a soluble state of the bowels, would advise one teaspoonful in a wineglass of water three times a day of the following: ℞. Fraxinus Americana cortex ℥i; Asclepias SyriacaRadix ℥v.; Brandy oi.

DEC. 22.—Charlotte feels a great deal better in every respect; appetite much improved, increased in strength and flesh; had her courses last week; they were attended with much less pain. Examination with the speculum; cervix less engorged, and Leucorrhea about the same. The above prescription continued with the addition of ten drops of Balsam Copaiba to each dose.

DECEMBER 4th.—*Chronic Pneumonia.*—Penny, a colored woman, aged 43. Has been troubled with a cough and hoarseness for 15 years. She takes cold on the slightest change in the weather or least exposure; becomes so hoarse in a few minutes, that she cannot be heard above a whisper. Coughs almost incessantly for a few days, expectorates freely, a frosty mucus sometimes mixed with blood. Percussion is tolerably clear throughout the chest. Auscultation, however, reveals the existence of the mucous ronchus; the pulse quick and rather feeble; skin damp and flaccid, with some tendency to œdema.

*Treatment.*—Penny is too old, and her case is of too long standing to derive any benefit from general bleeding, but occasional cupping over the spine between the shoulders might be beneficial, but in this case our chief reliance is on stimulating expectorants—Seneka, Balsam Copaiba, ammoniac, tolu and spirits of turpentine, are regarded the most useful, but I would recommend Penny to take every third night a pill composed of Blue Mass, gr. ii; Pulv. opii gr. ¼, and a tea-spoonful of the following three times a day: ℞. Tar ℥i; Honey ʒiv; Rum oi; mix well by boiling bottle a few moments.

*Service of Dr. Logan.*

*Cutaneous Ulceration.*—Ellen, aged 13, was presented with extensive superficial ulceration of the arm, as the result of a slight abrasion of the skin some weeks before.

It was suggested that she might possibly be laboring under the scrofulous diathesis, but the plan of treatment (domestic) to which she had been subjected, was decidedly injudicious, and perhaps sufficient to account for the indisposition to heal, found in the part affected.

A considerable part of the treatment had been the frequent application of oily matter, in accordance with the popular idea of the value of ointments, which was condemned in the most decided manner, as exerting a most injurious influence upon the exudation thrown out as the basis of a new structure, producing a degeneration of the plastic matter, and thus preventing healthy granulation. The frequent washing, and exposure of the irritated surface to the air, might also have contributed in some degree, to the obstinacy of the affection. The proper treatment was the induction of a revulsive and cathartic action upon the intestinal canal by the administration of: ℞. Cream of Tartar; Calcine Magnesia, *aa* ʒi, and the maintenance of a soluble condition of the bowels, and in addition, as a local application: ℞. Hydrarg. Cum. Creta, ʒi; Creta Preparata, ℥ss; mix thoroughly; to be sprinkled over the affected part three or four times a day.

*Scrofula.*—Maria, a negro girl, six or seven years of age, was evidently laboring under scrofulous enlargement of the cervical glands, with a very singular manifestation of the same disease in a discharge from the vagina, and an eruption upon the labia and thighs, strongly resembling syphilis, and which would doubtless in an older subject have been pronounced of a venereal character. Scrofula was *hereditary* as well as *acquired*; we had the scrofulous Diathesis, and the development of scrofula from certain influences or circumstances surrounding individuals in those *not predisposed.*

In a pathological point of view, the disorder under consideration was a *blood* disease, and consisted essentially of a deficiency in the red corpuscles of the blood, and in a deficiency in quantity, and a want of the proper elaboration of the fibrin, the primary difficulty, found perhaps in the failure of the digestive and blood-making apparatus to properly perform their functions.

Among the external influences operating most actively in the production of scrofula, may be regarded *impure air*, and it is even thought, by high authority, that if there be any diseased condition that is strictly the product of impure air, it is scrofula ; but, whether this be true or not, there is sufficient ground for stating that the habitual inspiration of an impure atmosphere is one of the most efficient agents in the *development* of scrofula, in those predisposed to the disease.

The treatment recommended for this patient was based upon what was supposed to be the true pathological condition. Tonics, bitter and ferruginous, to increase the activity of the digestive organs, and to increase the red corpuscles of the blood. Cod Liver Oil, and a nutritive diet to furnish the materials and the conditions most favorable for the preparation of a more perfectly elaborated blood, an occasional cathartic, with cleanliness, and a pure air.

Besides these means, which might be regarded as indicated upon *scientific* grounds proper, we have what is called the *specific* treatment of scrofula, and though the lecturer was not at all favorable to the idea of specifics generally, it was admitted that great importance should be attached to the use of Iodine in this affection, the most astonishing results having been witnessed in his own experience. To derive the full benefit of any treatment, however, in this most obstinate malady, required great patience and perseverance, not only extending to months, but, in many instances, even to years. Mr. Pitts, laboring under chronic rheumatism, &c., again presented himself, decidedly improved, for whom a continuation of the same treatment was directed, with the addition of Iodide of Potassium, ʒi ; Vin. Colchici, ℨii; Water, ℥ii; mix; Sig. one teaspoonfull in a wine glass of water, three times a day.

Mr. Jenkins, with indigestion and constipated bowels, reports himself better, and in addition to the vegetable tonics formerly prescribed, he was directed to take one of the following pills each night: Rhubarb; Aloes; Castile Soap, aa ℨss; Simple Syrup, q. s.; mix and divide into 30 pills.

---

*Service of Dr. Jones.*

*Elephantiasis.*—William, a negro man about thirty-eight years old, of large stature, and apparently enjoying excellent health, (with one exception,) came before the class, and stated in substance, that about five years since and during the summer season his left leg began gradually to swell, and was for several weeks quite painful, and tender when pressed; that the sensation of pain partially subsided in the course of a few weeks, but that the enlargement remained or rather increased with each successive attack of what he termed the painful *spells*, which recurred every two or three months. About three years since the sense of feeling was greatly diminished in the diseased limb, that two years ago the swelling was greater than at any time before or since, and that shortly thereafter he was considerably relieved by medical treatment, an account of which treatment was obtained from a medical friend, and consisted mainly of Donovan's solution of Arsenic, &c., internally—the occasional application of Iodine ointment, and regular pressure by means of a roller bandage extending from the toes to the knee, together with the observance of regular habits, temperate living and rest from hard labor. He also stated that he was still subject to *bad spells* of pain and stiffness in the diseased leg, and that a calomel purge usually put him on foot in a few days after it was administered.

The patient's leg and foot is now much tumefied, indurated and rugosed, and about the ankle several deep fissures exist, immediately over and circumjacent to the external malleolar region there is a large cluster of soft fungous excrescences of a bronzed appearance, all of which symptoms in connection with the history of the case, induce the conclusion, that the disease is one of the varieties of Elephantiasis. Examples of this disease differing in variety and locality, are not unfrequent in the Antilles—it is sometimes called the Barbadoes leg. In Arabia, in Cayenne, Greece, India, Italy, and Java, it is not uncommon. The etiology of this disease is quite obscure. Some regard it as contagious, and some advocate its transmissibility by inheritance. It is also believed to have prevailed epidemically in parts of Europe, and endemically in hot climates.

Billy's case was regarded as presenting some of the features of two varieties, viz: Those described by Grecian and Arabian writers, the former of which appears to be mainly a turberculated affection of the integuments and not unfrequently indicative of general tuberculosis, the latter as a consequence of inflammation of the lymphatics and an altered condition of the subcutaneous cellular tissue. The prognosis in the case under consideration was deemed unfavorable—notwithstanding the relief afforded by previous treatment, to which allusion has been made—on the authority of writers. A number of remedies were enumerated, as having operated beneficially, among which were preparations of Iodine and Arsenic, Mezereon, Stillingia, &c. In cases of long standing, and those that had resisted the ordinary modes of treatment, it was suggested and recommended to make a few deep, but not too long incisions into certain portions of the affected parts, and then by the introduction of irritating substances or even the actual cautery, establish a disorganizing process that would remove in some degree, the morbid growths, and then depend mainly upon the recuperative efforts of the system to repair the breech with a more normal material. It was stated that this mode of treatment, had in a few instances, effected desirable results, and was worthy a more extended trial. Amputation was mentioned as a dernier resort in extreme, or incurable cases.

