# Errata

**Published:** 1859-03

**Authors:** 


### ERRATA.

February No., page 338, third line from top, for ℥v read ʒv—on same page, bottom line, for Honey ʒiv read ℥iv.

